# Fibrillar α-synuclein induces neurotoxic astrocyte activation via RIP kinase signaling and NF-κB

**DOI:** 10.1038/s41419-021-04049-0

**Published:** 2021-07-31

**Authors:** Tsui-Wen Chou, Nydia P. Chang, Medha Krishnagiri, Aisha P. Patel, Marissa Lindman, Juan P. Angel, Po-Lun Kung, Colm Atkins, Brian P. Daniels

**Affiliations:** Department of Cell Biology and Neuroscience, Rutgers University, Piscataway, NJ USA

**Keywords:** Cell death and immune response, Cell death in the nervous system, Astrocyte, Neuroimmunology, Parkinson's disease

## Abstract

Parkinson’s disease (PD) is a neurodegenerative disorder characterized by the death of midbrain dopamine neurons. The pathogenesis of PD is poorly understood, though misfolded and/or aggregated forms of the protein α-synuclein have been implicated in several neurodegenerative disease processes, including neuroinflammation and astrocyte activation. Astrocytes in the midbrain play complex roles during PD, initiating both harmful and protective processes that vary over the course of the disease. However, despite their significant regulatory roles during neurodegeneration, the cellular and molecular mechanisms that promote pathogenic astrocyte activity remain mysterious. Here, we show that α-synuclein preformed fibrils (PFFs) induce pathogenic activation of human midbrain astrocytes, marked by inflammatory transcriptional responses, downregulation of phagocytic function, and conferral of neurotoxic activity. These effects required the necroptotic kinases RIPK1 and RIPK3, but were independent of MLKL and necroptosis. Instead, both transcriptional and functional markers of astrocyte activation occurred via RIPK-dependent activation of NF-κB signaling. Our study identifies a previously unknown function for α-synuclein in promoting neurotoxic astrocyte activation, as well as new cell death-independent roles for RIP kinase signaling in the regulation of glial cell biology and neuroinflammation. Together, these findings highlight previously unappreciated molecular mechanisms of pathologic astrocyte activation and neuronal cell death with implications for Parkinsonian neurodegeneration.

## Introduction

Parkinson’s disease (PD) is the second most common neurodegenerative disease after Alzheimer’s disease [1]. Pathologically, PD is characterized by progressive loss of dopaminergic neurons in the substantia nigra pars compacta (SNpc), as well as axonal degeneration in the nigrostriatal pathway and reduction of dopamine inputs into the striatum [2]. Though the pathogenesis of PD is poorly understood, growing evidence implicates aggregated forms of the protein α-synuclein as an etiologic agent of Parkinsonian neurodegeneration [3, 4]. Misfolding of soluble α-synuclein monomers leads to the formation of insoluble aggregates that exert neurotoxic and inflammatory activity, contributing to the neuronal death and degeneration observed in PD and other synucleinopathies [5, 6].

While the impact of aggregated α-synuclein on neurons has been extensively described, roles for α-synuclein in astrocytes are comparatively poorly understood. Astrocytes serve key homeostatic functions, including promoting neurite outgrowth and phagocytosing cellular debris [7]. However, the activation and proliferation of astrocytes has been associated with both protective and pathologic functions in many neurodegenerative diseases, including PD [8, 9], Alzheimer’s disease [10], Huntington’s disease [11], amyotrophic lateral sclerosis [12, 13], and others [7, 14–22]. Previous work has described at least two putative subtypes of reactive astrocytes, termed “A1,” which are pro-inflammatory and neurotoxic, and “A2,” which are generally anti-inflammatory and neurotrophic [18]. These activation states have been distinguished by distinct transcriptional signatures and functional profiles, including the propensity to induce cell death in neurons (a marker of A1 astrocyte activity). While aggregated α-synuclein has been shown to contribute to inflammatory astrocyte activation [23–25], the transcriptional and functional consequences of this effect have not been fully established. Moreover, the molecular mechanisms that promote inflammatory astrocyte activation downstream of pathogenic α-synuclein species are poorly defined.

During neurodegeneration, inflammatory signals induce multiple forms of programmed cell death in susceptible neural cells, including both apoptosis and necroptosis [26–30]. Necroptosis is a form of programmed cell death mediated by receptor-interacting protein kinases-1 (RIPK1) and −3 (RIPK3). These kinases coordinate activation of the executioner pseudokinase mixed lineage kinase domain-like protein (MLKL), which permeabilizes cell membranes, resulting in necrotic cell death [31, 32]. However, aside from necroptosis, several groups have recently described pleiotropic, cell death-independent functions for RIPK signaling [33–39]. We and others have shown that, in neurons, RIPK activation can initiate inflammatory transcriptional responses without inducing MLKL oligomerization and host cell death following neurotropic viral infection [33, 34, 40]. However, whether necroptosis-independent functions for RIPK signaling are relevant during sterile neurodegenerative diseases requires further investigation. Moreover, whether RIPK signaling promotes inflammatory signaling in nonneuronal cells of the central nervous system (CNS), such as astrocytes, has yet to be fully addressed.

In this study, we sought to define the impact of aggregated α-synuclein on astrocyte activation state. We show that α-synuclein preformed fibrils (PFFs) induce robust inflammatory transcriptional signaling in human midbrain astrocytes, including transcripts associated with both the putative A1 and A2 astrocyte activation states. Functional analyses demonstrated that α-synuclein PFFs conferred neurotoxic activity in midbrain astrocytes, while diminishing homeostatic phagocytic activity. Each of these transcriptional and functional outcomes required the inflammatory transcription factor NF-κB. Importantly, NF-κB activation and subsequent inflammatory transcription and neurotoxic activation could be rescued via pharmacological blockade of RIPK1 and RIPK3 signaling, while MLKL was dispensable for these effects, which occurred in the absence of astrocytic cell death. These data identify a previously unknown necroptosis-independent function for RIPK signaling in promoting a neurotoxic activation state in astrocytes following exposure to a pathogenic species of α-synuclein.

## Materials and methods

### α-synuclein preformed fibrils

Purified human α-synuclein monomers were purchased from Proteos, Inc. (Kalamazoo, MI, #RP-003) and were used to generate PFFs according to established protocols [41]. Briefly, monomers were diluted to a concentration of 5 mg/mL with PBS and agitated on a thermomixer at 1000 RPM at 37 °C for 7 days. Fibrilization was confirmed by measuring fluorescence in the presence of thioflavin T (Supplementary Fig. 1), as previously described [42]. Briefly, 95 µL of thioflavin T (25 mM) diluted in PBS was mixed with 2.5 µL of lab-generated PFF stocks or monomeric control stocks. Samples were mixed for 15 m at room temperature. Fluorescence was measured using a SpectraMax iD3 plate reader (Molecular Devices, San Jose, CA) at excitation 450 nm, emission 500 nm. PFF stocks were diluted to a concentration of 100 μg/mL in 1x HBSS buffer followed by three 10 s pulses of sonication using a 1/8*″* probe equipped QS5 Sonicator (Covaris, Woburn, MA) prior to use. For cell culture experiments, freshly sonicated PFFs were used at 0.1 μg/mL.

### Inhibitors

BAY 11–7085 (#1743), SR 11302 (#2476), and Pyridone 6 (#6577) were purchased from Tocris Bioscience (Bristol, UK). JSH-23 was purchased from Selleck Chemicals (#S7351, Houston, TX). GSK963 (#SML2376), GSK872 (#530389), necrosulfonamide (#480073), and Z-VAD-FMK (#627610) were purchased from Millipore Sigma (Burlington, MA). All inhibitors were solubilized in DMSO. BAY 11–7085, SR 11302, and Pyridone 6 were used at a final concentration of 100 μM for cell culture treatments. JSH-23 was used at 50 μM. GSK 963 and GSK 872 were used at 1 μM. Necrosulfonamide was used at 10 μM. Z-VAD-FMK was used at 5 μM.

### Human astrocyte and neuronal cultures

Primary human midbrain astrocytes (#1850) were obtained from ScienCell Research Laboratories (Carlsbad, CA) and cultured in astrocyte media (AM, #1801), supplemented with 2% heat-inactivated fetal bovine serum (#0010), astrocyte growth supplement (#1852), and penicillin/streptomycin cocktail (# 0503). Cells were cultured in poly-l-lysine coated T75 flasks. Human neuronal cells SH-SY5Y (ATCC, Manassas, VA, #CRL-2266) were cultured in DMEM medium (VWR, Radnor, PA, #0101–0500) supplemented with 10% FBS (Gemini Biosciences West Sacramento, CA, #100–106), nonessential amino acids (Hyclone, #SH30138.01), HEPES (Hyclone #30237.01), penicillin, streptomycin, and antifungal (Gemini Biosciences #400–110, #100–104). SH-SY5Y cells were propagated in T75 flasks prior to the differentiation process. Low passage stocks (less than 15 passages) were used for differentiation throughout the manuscript. Cells were regularly screened for mycoplasma contamination.

### SH-SY5Y differentiation

SH-SY5Y neuroblastoma cells were differentiated into mature neuron-like cells by treating with retinoic acid (4 μg/mL; Sigma-Aldrich, St. Louis, MO, #R2625) and BDNF (25 ng/mL, Sigma-Aldrich, #B3795) diluted in DMEM supplemented with 2% heat-inactivated fetal bovine serum (FBS, Gemini Biosciences, West Sacramento, CA, #100–106), nonessential amino acids (1x; HyClone, #SH30238.01), HEPES buffer (10 mM; HyClone, #SH30237.01), l-Glutamine, penicillin, streptomycin (Gemini Biosciences, #400–110), and antifungal amphotericin B (Gemini Biosciences, #100–104). Differentiated SH-SY5Y cultures were used for experiments 7 days post-differentiation.

### Cell death and viability assay

Cell viability was assessed with the CellTiter-Glo Luminescent Cell Viability Assay kit (Promega, Madison, WI, #G7573), according to the manufacturer’s instructions. Luminescence signal was read with a SpectraMax iD3 plate reader (Molecular Devices, San Jose, CA).

### Caspase 3/7 activity assay

Caspase 3/7 activity was measured using a chromogenic DEVD cleavage assay according to the manufacturer’s instructions (R&D Systems, Minneapolis, MN, #K106-100).

### Quantitative real-time PCR

Total RNA from cultured cells was isolated with Qiagen RNeasy mini extraction kit (Qiagen, Valencia, CA, #74106) following the manufacturer’s protocol. RNA concentration was measured with a Quick Drop device (Molecular Devices, San Jose, CA). cDNA was subsequently synthesized with qScript cDNA Synthesis Kit (Quantabio, Beverly, MA, #95047). qRT-PCR was performed with SYBR Green Master Mix (Bio-Rad, Hercules, #CA1725125) using a QuantStudio5 instrument (Applied Biosystems, Foster City, CA). Cycle threshold (CT) values for analyzed genes were normalized to CT values of the housekeeping gene *18* *S* (CT_Target_ − CT_18S_ = ΔCT). Data were further normalized to baseline control values (ΔCT_experimental_ − ΔCT_control_ = ΔΔCT (DDCT). Primers were designed using Primer3 (https://bioinfo.ut.ee/primer3/) against human genomic sequences. A list of all primer sequences in our study appears in Supplementary Table 1.

### Immunocytochemistry

For imaging experiments, cells were grown on poly-d-lysine coated coverslips (Neuvitro, Vancouver, WA, #GG-12-PDL). Following experimental treatments, cells were fixed in 4% paraformaldehyde for 15 min, followed by three washes in 1x PBS, followed by incubation in blocking solution (10% goat serum, Gibco, Waltham, MA, #16210 and 0.1% Triton X-100) for 30 min at room temperature. Cells were then incubated in primary antibody (rabbit-anti-p65/RELA; 2 ug/mL; ThermoFisher, Waltham, MA, #10745-1-AP) diluted in blocking solution for 1 h. After three 15 min washes in 1x PBS, coverslips were incubated in secondary antibody (goat-anti-rabbit IgG conjugated Alexa Fluor 594; 2 ug/mL; Invitrogen Waltham, MA, #A32740) and nuclear stain (DAPI; 10 ug/mL; Biotium, Fremont, CA, #40043) for 15 min at room temperature, followed by another series of washes with 1x PBS. Coverslips were then mounted using ProLong Diamond Antifade Mountant (Invitrogen, #P36931) onto slides. Images were acquired with an Airyscan fluorescent confocal microscope (Carl Zeiss LSM 800).

### TUNEL assay

TUNEL was performed using a standard kit according to the manufacturer’s protocol (TMR In Situ Cell Death Detection Kit, Sigma-Aldrich, #12156792910) in combination with nuclear staining using DAPI (10 ug/mL; Biotium, Fremont, CA, #40043). Images were captured using a 20x objective. The numbers of TUNEL positive nuclei in each image were counted by a blinded operator.

### Colocalization analysis

For each coverslip, three regions with matched cell density were captured with the 63x objective. p65 colocalization with DAPI was quantified using the Colocalization Colormap plugin in ImageJ software (National Institute of Health, Bethesda, MD). The plugin calculates normalized mean deviation product, an index of correlation between pixels. Fisher’s Z transformation was applied to the index of correlation prior to comparison.

### Nuclear protein extraction

Primary human midbrain astrocytes were cultured to confluency. Cells were washed twice with PBS followed by 5 min incubation in cold 5 mM EDTA. Cells were then scraped into 15 mL conical tubes and centrifuged for 5 min at 1000 rpm. Nuclear extraction was performed using a standard kit according to the manufacturer’s protocol (Nuclear Extraction Kit, Abcam, Cambridge, MA, #ab113474).

### NF-κB p65 transcription factor assay

Protein concentrations of nuclear extracts were determined using BCA assay (ThermoFisher, #23227), according to the manufacturer’s instructions. Equal amounts of protein were then processed through an ELISA-based kit for detecting p65 (NF-κB p65 Transcription Factor Assay Kit, Abcam, Cambridge, MA, #ab133112). Absorbance at 450 nm was read with SpectraMax iD3 plate reader (Molecular Devices, San Jose, CA).

### Flow cytometric analysis of phagocytosis

Differentiated SH-SY5Y neuronal cells were labeled with CSFE, according to the manufacture protocol (Millipore Sigma, Burlington, MA, #SCT110) and lysed using repeated freeze-thaw cycles to generate labeled debris. Unlabeled neuronal debris was used as staining control. Neuronal debris was stored at −80 °C until needed. To detect phagocytosis, CSFE-labeled neuronal debris was added to astrocyte cultures at a ratio of 1:100 for 24 h. Un-phagocytosed neuronal debris was washed away with 1XPBS, and astrocytes were harvested with 5 mM EDTA followed by scraping of adherent cells. Astrocytes were stained with Zombie NIR at 1:1000 in 1XPBS according to the manufacturer protocol (BioLegend, San Diego, CA, #423105), followed by fixation in 1% paraformaldehyde. Data collection and analysis were performed using a Northern Lights flow cytometer (Cytek, Fremont, California) and FlowJo software (FlowJo LLC, Ashland, OR).

### Statistical analysis

Normally distributed data were analyzed using appropriate parametric tests: two-way analysis of variance (ANOVA) with Sidak’s correction for multiple comparisons was performed using GraphPad Prism Software v8 (GraphPad Software, San Diego, CA). *P* < 0.05 was considered statistically significant. Analysis of publicly available microarray data was performed in GEO2R and the GO Enrichment Analysis tool [43]. Corrected *p* values (false discovery rate) were determined using the Benjamini & Hochberg procedure. All data points represent biological replicates unless otherwise noted.

## Results

### α-synuclein PFFs induce NF-κB-dependent transcriptional activity associated with astrocyte activation

To assess the impact of α-synuclein aggregates on astrocyte activation state, we treated primary cultures of human midbrain astrocytes with α-synuclein PFFs, which have been extensively shown to induce inflammatory activation and seed aggregation of endogenous α-synuclein both in vitro and in vivo [41, 44, 45]. To profile the astrocyte activation state following this treatment, we performed a qPCR screen of transcripts previously associated with the putative A1 (neurotoxic) and A2 (neurotrophic) astrocyte activation states [18], along with other general markers of inflammatory activity. To further characterize the nature of the PFF-induced transcriptional response, we performed these experiments in the presence of inhibitors of three major inflammatory transcriptional pathways: BAY 11–7085 (BAY) is an irreversible inhibitor of IκB kinase (IKK), thereby blocking NF-κB activation; Pyridone 6 (PYR) is a pan-JAK inhibitor, thereby blocking signaling through STAT family transcription factors; and SR 11302 (SR) is an inhibitor of the transcription factor AP1.

**Fig. 1:**
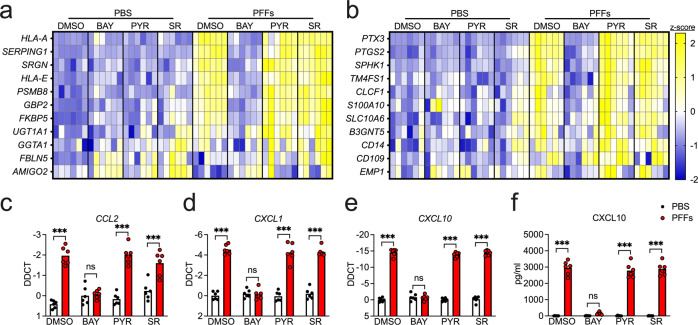
α-synuclein PFFs induce NF-κB-dependent transcriptional activation in human midbrain astrocytes. **a–f** Primary human midbrain astrocyte cultures were treated for 24 h with PFFs or PBS control solution. Cultures were pretreated (30 min) with inhibitors of NF-κB (BAY), JAK/STAT (PYR), or AP1 (SR) signaling prior to the addition of PFFs. Levels of indicated A1-associated transcripts (**a**), A2-associated transcripts (**b**), and inflammatory chemokines (**c–e**) were measured using qRT-PCR. **f** Levels of CXCL10 protein in culture supernatants were measured via ELISA. Data in (**a**) and (**b**) were normalized and z-transformed summaries of the qPCR data that appears in Supplementary Figs. 2 and 3, represented via heatmaps. ns not significant, *p < 0.05, **p < 0.01, ***p < 0.001. Bars represent group means. *n* = 6 independent replicates for all experiments.

Overnight treatment with PFFs induced increased expression of 8 of 11 A1-associated transcripts in our screen (Fig. 1a and Supplementary Fig. 2a–h), including *HLA-A*, *SERPING1*, *SRGN*, *HLA-E*, *PSMB8*, *GBP2*, *FKBP5*, and *UGT1A1*, while expression of *GGTA1*, *FBLN5*, and *AMIGO2* was not significantly impacted (Supplementary Fig. 2i–k). Notably, inhibition of NF-κB signaling with BAY rescued the upregulation of all eight A1-associated transcripts, while inhibition of JAK/STAT or AP1 signaling had minimal effect. Surprisingly, however, PFF treatment also upregulated 5 of 11 A2-associated genes in our study, including *PTX3*, *PTGS2*, *SPHK1*, *TM4FS1*, and *CLCF1* (Fig. 1b and Supplementary Fig. 3a–e), while *S100A10*, *SLC10A6*, *B3GNT5*, *CD14*, *CD109*, and *EMP1* each showed various degrees of upregulation that did not reach statistical significance (Supplementary Fig. 3f–k). PFF treatment also increased the expression of a number of inflammatory chemokines, including *CCL2*, *CXCL1*, *CXCL10* (Fig. 1c–e). The upregulation of CXCL10 was also detected at the protein level via ELISA (Fig. 1f). In all cases, blockade of NF-κB signaling, but not JAK/STAT or AP1 signaling, prevented upregulation of reactive astrocyte genes. Importantly, treatment of midbrain astrocytes with a matched concentration of α-synuclein monomers did not induce expression of either A1- or A2- associated transcripts (Supplementary Fig. 4a, b). Inflammatory responses to PFFs were also independent of serum concentrations in astrocyte culture media, as we observed essentially identical results in astrocytes grown in serum-free medium (Supplementary Fig. 5a–l). Together, these data suggest that α-synuclein PFFs strongly induce an NF-κB-dependent transcriptional response that includes a broad array of genes associated with astrocyte activation and inflammatory activity.

### α-synuclein PFFs induce both expression and activation of NF-κB signaling elements

**Fig. 2:**
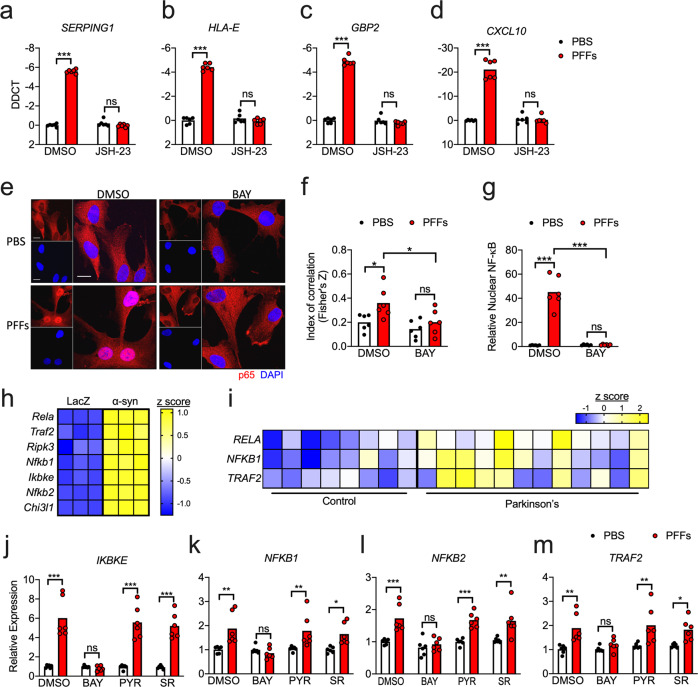
α-synuclein induces NF-kB expression and activation in astrocytes. **a–d** qRT-PCR analysis of indicated genes in human midbrain astrocyte cultures treated following 24 h PFF treatment ± cotreatment with JSH-23. **e** Confocal microscopy of NF-κB component p65 (red) and nuclei (DAPI, blue) in human midbrain astrocytes following 2 h PFF treatment ± cotreatment with BAY. Scale bars = 20 μm. **f** Colocalization of red and blue signal in (**e**) is quantified as Fisher’s Z transformed index of correlation. **g** Nuclear fractions were isolated from human midbrain astrocyte cultures following 2 h PFF treatment ± cotreatment with BAY. Relative levels of p65 in nuclear fractions were quantified using ELISA. **h**, **i** Secondary analysis of microarray profiling of (**h**) rat astrocytes treated with conditioned neuronal medium containing LacZ or α-synuclein (GSE11574) or **i** human postmortem substantia nigra samples from patients with PD or healthy controls (GSE26927). Z scores were calculated for genes under the GO term 0038061 and those exhibiting significantly differential expression between groups (corrected *p* value < 0.05) are displayed in the respective heatmaps. **j–m** qRT-PCR analysis of indicated genes in human midbrain astrocyte cultures treated following 24 h PFF treatment ± cotreatment with indicated inhibitors. ns not significant, *p < 0.05, **p < 0.01, ***p < 0.001. Bars represent group means. *n* = 6 independent replicates for all experiments.

We next sought to more thoroughly assess the impact of α-synuclein PFFs on NF-κB activation in astrocytes. While BAY blocks NF-κB signaling by inhibiting upstream IKK activity, we next tested whether direct blockade of NF-κB nuclear translocation would impact PFF-mediated gene expression using the inhibitor JSH-23, which also rescued the induction of several reactive astrocyte genes (Fig. 2a–d). To directly confirm that PFFs induced NF-κB activation, we performed confocal microscopy to visualize nuclear translocation of the NF-κB component p65. PFF treatment induced robust accumulation of p65 in the nucleus, as indicated by enhanced colocalization of p65 signal with nuclear DAPI staining (Fig. 2e, f). Importantly, treatment with BAY completely blocked this effect, confirming that IKK inhibition effectively blocked NF-κB activation in our experiments. As a secondary confirmation of NF-κB activation, we extracted nuclear fractions from astrocytes following treatment with PFFs or PBS control and performed ELISA to detect p65. These experiments also revealed a significant accumulation of nuclear p65 that was completely blocked by cotreatment with BAY (Fig. 2g).

We also questioned whether PFF treatment might influence NF-κB activity via upregulation of NF-κB signaling elements. To answer this, we first turned to two publicly available transcriptomic databases. In one study, Lee and colleagues [24] exposed primary rat astrocytes to a conditioned medium from a human neuroblastoma cell line (SH-SY5Y) that had been transduced to express either α-synuclein or a LacZ control. Secondary analysis of microarray data from this study revealed that exposure to α-synuclein-containing conditioned medium significantly upregulated many genes associated with the “NF-κB signaling” gene ontology term (GO:0038061), including *Rela* (p65), *Traf2*, *Ripk3*, *Nfkb1*, *Ikbke*, *Nfkb2*, and *Chi3l1* (Fig. 2h). We performed a similar analysis on a dataset originally published by Durrenberger and colleagues [46], who reported transcriptomic profiles of postmortem samples of substantia nigra tissues from 12 PD patients and eight healthy controls. Secondary analysis of this dataset revealed significant upregulation of three genes associated with NF-κB signaling, including *RELA*, *NFKB1*, and *TRAF2* (Fig. 2i). In our own experimental system, we saw that treatment of human midbrain astrocytes with PFFs also significantly upregulated expression of *IKBKE*, *NFKB1*, *NFKB2*, and *TRAF2* (Fig. 2j–m). Notably, the induction of these genes was blocked by BAY, suggesting that PFFs may induce feed-forward amplification of NF-κB signaling. Together, these data confirm that α-synuclein induces both expression and activation of NF-κB in astrocytes and that enhanced expression of NF-κB signaling elements in the substantia nigra is a feature of PD in human patients.

### α-synuclein PFFs induce neurotoxic activity in midbrain astrocyte cultures

**Fig. 3:**
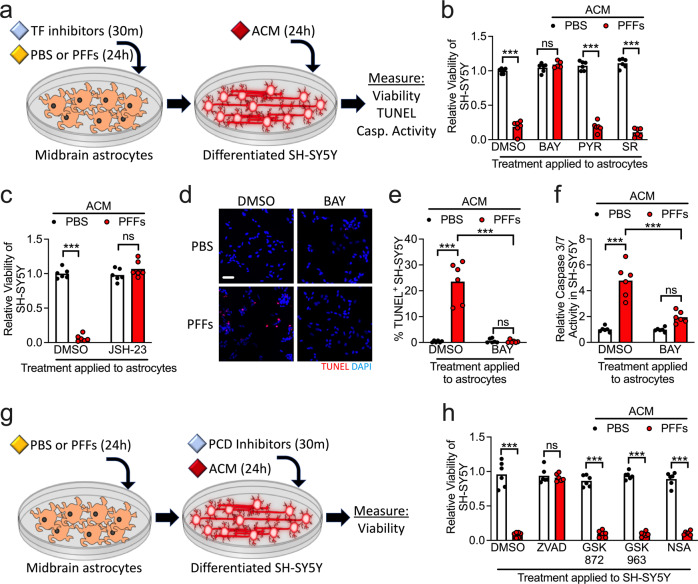
α-synuclein PFFs induce neurotoxic activity in midbrain astrocytes. **a–f** Primary human midbrain astrocytes were treated with transcription factor (TF) inhibitors and/or PFFs, as indicated. After 24 h, an astrocyte conditioned medium (ACM) was applied (1:1) to differentiated SH-SY5Y cultures for 24 h followed by endpoint analyses. **b**, **c** Viability of SH-SY5Y cells following treatment with ACM derived from astrocyte cultures treated with the indicated inhibitors was measured via ATP-luciferase assay (Cell Titer Glo). **d**, **e** Cell death of SH-SY5Y cells following 24 h ACM treatment was detected via TUNEL (**d**) and quantified as a percentage of TUNEL^+^ nuclei (**e**). **f** Levels of Caspase 3/7 activity in SH-SY5Y cells following treatments as in (**a**) was measured using a chromogenic DEVD-cleavage assay. **g** Modification of experimental setup in (**a**), in which SH-SY5Y cultures were pretreated with programmed cell death (PCD) inhibitors for 30 min prior to addition of ACM. **h** Viability of SH-SY5Y cells following treatment with ACM and PCD inhibitors as in (**g**) was measured via ATP-luciferase assay (Cell Titer Glo). ns not significant, *p < 0.05, **p < 0.01, ***p < 0.001. Bars represent group means. *n* = 6 independent replicates for all experiments.

Neuroinflammatory astrocytes, generally, have been reported to induce programmed cell death in neurons, thereby contributing to disease pathogenesis. To test if α-synuclein PFFs could confer neurotoxic activity to astrocytes, we treated midbrain astrocyte cultures with PFFs or PBS control in the presence of transcription factor pathway inhibitors (Fig. 3a). We then collected astrocyte conditioned medium (ACM) from these cultures and added it to cultures of differentiated SH-SY5Y neuroblastoma cells at a 1:1 ratio with a normal culture medium. We observed that 24 h treatment with ACM derived from astrocyte cultures treated with PFFs significantly reduced the viability of SH-SY5Y cultures, as assessed via ATP luciferase assay (Fig. 3b). Blockade of NF-κB signaling in astrocytes with BAY rescued this neurotoxic activity, while PYR and SR treatment did not. We saw similarly that NF-κB inhibition with JSH-23 also prevented neurotoxic activity in astrocytes following PFF treatment (Fig. 3c). As a secondary confirmation of cell death in SH-SY5Y cultures, we performed terminal deoxynucleotidyl transferase dUTP nick end labeling (TUNEL) to detect DNA damage associated with programmed cell death, which revealed a significant increase in TUNEL^+^ nuclei in SH-SY5Y cultures treated with ACM derived from PFF-treated astrocytes (Fig. 3d, e). However, the treatment of astrocytes with BAY prevented this effect. Together, these data suggest that α-synuclein PFFs stimulate neurotoxic activation in midbrain astrocytes in an NF-κB-dependent manner. Importantly, we confirmed that cell death in our study could not be explained by exposure to residual α-synuclein PFFs found in ACM samples, as direct treatment of SH-SY5Y with PFFs did not impact their viability within the timeframe of our experiments (Supplementary Fig. 6).

While apoptosis is the most commonly reported form of neuronal cell death during Parkinsonian neurodegeneration, other cell death modalities, including necroptosis, have also been reported [27, 47]. We questioned which cell death modality was induced in differentiated SH-SY5Ycells in our experiments. We first used a DEVD-cleavage assay to measure levels of executioner caspase (caspase 3 and caspase 7) activity, which revealed that ACM derived from PFF-treated astrocytes induced robust Caspase 3/7 activity in SH-SY5Y cultures, and that this effect was blocked when astrocytes were cotreated with BAY (Fig. 3f). To more carefully determine if caspase-dependent apoptosis was occurring, we modified our treatment paradigm such that, prior to the addition of ACM, SH-SY5Y cultures were pretreated with inhibitors of programmed cell death, including the pan-caspase inhibitor zVAD-FMK (zVAD), the RIPK3 inhibitor GSK872, the RIPK1 inhibitor GSK963, or the MLKL inhibitor necrosulfonamide (NSA) (Fig. 3g). Blockade of caspase signaling with zVAD completely rescued cell death in SH-SY5Y cultures following treatment with ACM derived from PFF-treated astrocytes, while inhibiting of necroptosis signaling components (RIPK1, RIPK3, or MLKL) did not (Fig. 3h). These data suggest that the neurotoxic activity conferred by PFFs in astrocytes induces apoptosis rather than necroptosis in SH-SY5Y cells.

### α-synuclein PFFs reduce homeostatic phagocytic activity in astrocytes

**Fig. 4:**
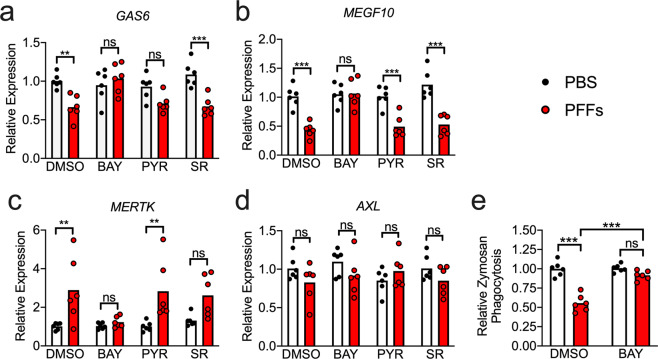
α-synuclein PFFs reduce phagocytic activity in midbrain astrocytes. **a–e** Primary human midbrain astrocyte cultures were treated for 24 h with PFFs or PBS control solution. Cultures were pretreated (30 min) with inhibitors of NF-κB (BAY), JAK/STAT (PYR), or AP1 (SR) signaling prior to the addition of PFFs. **a–d** Levels of indicated phagocytosis-associated transcripts were measured using qRT-PCR. **e** Twenty-four hours following PFF or PBS treatment, relative rates of phagocytosis were assessed by measuring uptake of fluorescently labeled zymosan. ns not significant, *p < 0.05, **p < 0.01, ***p < 0.001. Bars represent group means. *n* = 6 independent replicates for all experiments.

Neurotoxic astrocytes have been shown to downregulate key homeostatic functions, including phagocytosis. We thus questioned whether α-synuclein PFFs would perturb the phagocytic function of midbrain astrocytes. We treated astrocyte cultures with PFFs for 24 h in the presence of transcription factor pathway inhibitors and measured the expression of key genes known to be involved in astrocytic phagocytosis. PFF treatment downregulated expression of *GAS6* (Fig. 4a), which encodes an important opsonin for phagocytosis of apoptotic cells, as well as *MEGF10* (Fig. 4b), which encodes a receptor for C1q, an important opsonin in the classical complement pathway. In contrast, expression of *MERTK*, a GAS6 receptor, was increased by PFF treatment (Fig. 4c), which may represent a compensatory response to decreased GAS6 expression. Expression of *AXL*, which encodes an additional GAS6 receptor, was not impacted (Fig. 4d). BAY treatment prevented all PFF-induced changes to phagocytic gene expression, while PYR and SR had marginal or no impact. To determine whether changes to phagocytic gene expression actually impacted phagocytic activity, we measured uptake of fluorescently labeled zymosan, a yeast-associated glucan, in astrocyte cultures following 24 h treatment with PFFs and/or BAY (or respective controls). PFF-treated astrocytes phagocytosed significantly less zymosan compared to control cultures, and this effect was blocked in the presence of BAY (Fig. 4e). These data suggest that, in addition to inducing inflammatory gene transcription and neurotoxic activity, NF-κB activation downstream of PFF treatment reduces the homeostatic phagocytic capacity of astrocytes.

### α-synuclein PFFs induce RIPK-dependent transcriptional activation in astrocytes, independently of necroptosis

**Fig. 5:**
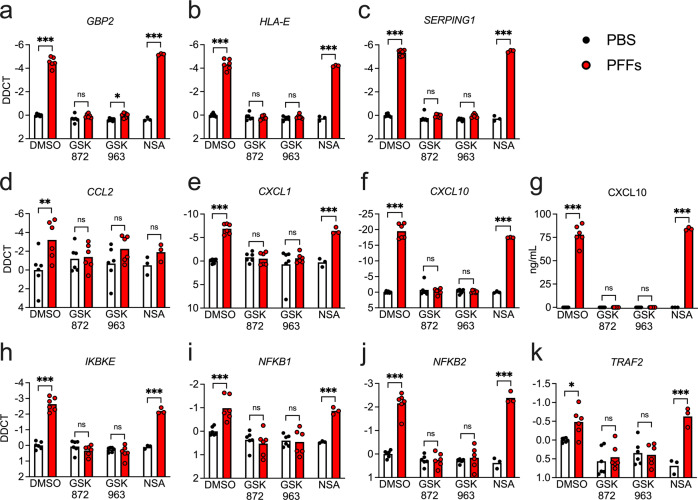
α-synuclein PFF-mediated transcriptional activation in astrocytes requires RIPK1 and RIPK3. **a–j** Primary human midbrain astrocyte cultures were treated for 24 h with PFFs or PBS control solution. Cultures were pretreated (30 min) with inhibitors of RIPK3 (GSK872), RIPK1 (GSK963), or MLKL (NSA) signaling prior to the addition of PFFs. **a–f**, **h–k** Levels of indicated transcripts were measured using qRT-PCR. **g** Levels of CXCL10 protein in culture supernatants were measured via ELISA. ns not significant, *p < 0.05, **p < 0.01, ***p < 0.001. Bars represent group means. *n* = 3–6 independent replicates for all experiments.

While our data clearly identified NF-κB as a molecular driver of astrocyte activation in our system, the upstream inputs into this pathway remained unclear. We previously described a role for RIPK signaling in inflammatory transcriptional activation in neurons that was independent of necroptotic cell death [34]. Moreover, RIPK signaling is a known activator of the NF-κB pathway [48–51]. We thus questioned whether RIPK signaling was required for PFF-mediated astrocyte activation. Treatment of midbrain astrocyte cultures with PFFs for 24 h in the presence of inhibitors of either RIPK3 or RIPK1 revealed that both kinases were required for induction of the PFF-mediated transcriptional response, including genes associated with astrocyte activation (Fig. 5a–c) and inflammatory chemokines (Fig. 5d–f). Blockade of RIPK signaling also prevented the induction of CXCL10 protein expression, as confirmed by ELISA (Fig. 5g). We also observed that inhibition of both RIPK3 and RIPK1 prevented transcriptional induction of NF-κB associated genes (Fig. 5h–k). Notably, these effects were independent of necroptosis, as inhibition of MLKL had no effect on PFF-mediated transcriptional activation (Fig. 5a–f, h–k), nor did it impact protein expression of CXCL10 (Fig. 5g). Moreover, neither PFFs nor any of the inhibitors in our study induced detectable levels of cell death in astrocytes (Supplementary Fig. 7), further confirming that necroptosis was not the source of transcriptional activation downstream of astrocytic RIPK signaling. Blockade of caspase signaling was also not required for these effects, as treatment with zVAD prior to PFF exposure had no effect on gene expression (Supplementary Fig. 8a). These data suggest that RIPK1 and RIPK3 engage inflammatory transcriptional activity in astrocytes following PFF treatment via a cell death-independent mechanism.

### RIPK signaling is required for NF-κB activation downstream of α-synuclein PFFs

**Fig. 6:**
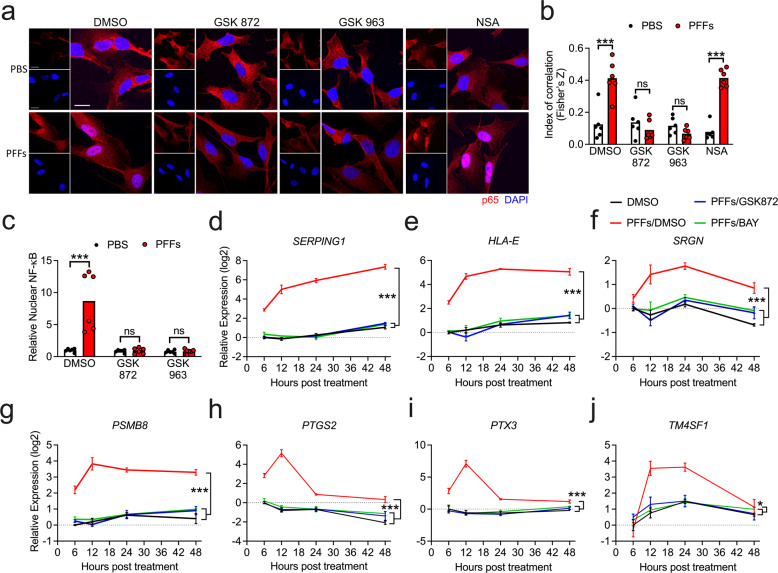
RIPK signaling is required for NF-κB activation downstream of α-synuclein PFFs. **a** Confocal microscopy of NF-κB component p65 (red) and nuclei (DAPI, blue) in human midbrain astrocytes following 2 h PFF treatment ± cotreatment with indicated inhibitors. Scale bars = 50 μm. **b** Colocalization of red and blue signal in (**a**) is quantified as Fisher’s Z transformed index of correlation. **c** Nuclear fractions were isolated from human midbrain astrocyte cultures following 2 h PFF treatment ± cotreatment with indicated inhibitors. Relative levels of p65 in nuclear fractions were quantified using ELISA. **d–j** Primary human midbrain astrocyte cultures were treated with PFFs or PBS control solution. Cultures were pretreated (30 min) with inhibitors of RIPK3 (GSK872) or NF-κB (BAY) signaling prior to the addition of PFFs. Levels of indicated transcripts were measured at indicated time points using qRT-PCR. ns not significant, *p < 0.05, **p < 0.01, ***p < 0.001. Bars represent group means. *n* = 6 independent replicates in (**a–c**). *n* = 3 independent replicates in (**d–j**).

We next confirmed that RIPK signaling was required for NF-κB activation in PFF-treated astrocytes. Confocal microscopic analysis of p65 expression revealed that blockade of either RIPK1 or RIPK3 was sufficient to prevent nuclear accumulation of p65, while blockade of MLKL had no effect (Fig. 6a, b). We observed similar findings following the detection of p65 in isolated nuclear fractions using ELISA (Fig. 6c). To further assess the relatedness of the RIPK3- and NF-κB-dependent transcriptional responses to PFFs, we performed a more thorough time-course analysis of gene expression associated with astrocyte activation in the presence of RIPK3 and NF-κB inhibitors. Remarkably, blockade of both molecules had essentially identical effects, as both inhibitors completely blocked the upregulation of astrocyte activation-associated genes following PFF treatment (Fig. 6d–j). Notably, however, these experiments revealed that the putative A1 genes *SERPING1*, *HLA*-*E*, *SRGN*, and *PSMB8* remained highly expressed up to 48 h following PFF exposure (Fig. 6d–g), while the A2 genes *PTGS2*, *PTX3*, and *TM4SF1* exhibited a distinct profile of robust early expression that largely resolved by 48 h (Fig. 6h–j). While RIPK3 and NF-κB did not appear to influence this temporally distinct expression pattern, these data suggest that the nature of astrocyte activation following exposure to α-synuclein aggregates may vary significantly over time.

### RIPK signaling is required for functional markers of astrocyte activation downstream of α-synuclein PFFs

**Fig. 7:**
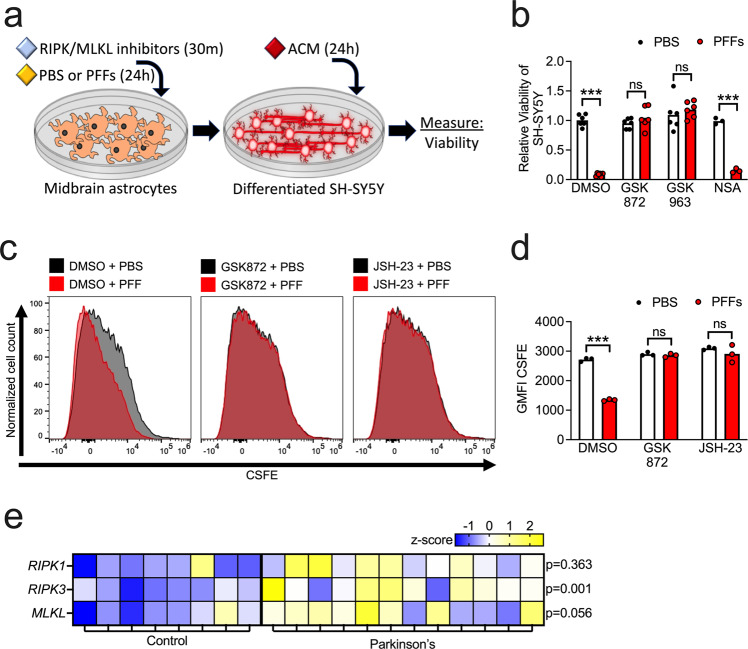
RIPK signaling is required for functional markers of astrocyte activation downstream of α-synuclein PFFs. **a** Primary human midbrain astrocytes were treated with RIPK inhibitors and/or PFFs, as indicated. After 24 h, an astrocyte conditioned medium (ACM) was applied (1:1) to differentiated SH-SY5Y cultures for 24 h followed by endpoint analyses. **b** Viability of SH-SY5Y cells following treatment with ACM derived from astrocyte cultures treated with the indicated inhibitors was measured via ATP-luciferase assay (Cell Titer Glo). **c**, **d** Primary human midbrain astrocyte cultures were treated with PFFs or PBS control solution. Cultures were pretreated (30 min) with inhibitors of RIPK3 (GSK872) or NF-κB (JSH-23) signaling prior to the addition of PFFs. Following this, CSFE-labeled neuronal debris was added for 24 h. Uptake was measured via flow cytometric analysis of CSFE signal in astrocytes. Data were represented as histograms (**c**) and quantified using mean geometric fluorescence intensity (GMFI) values (**d**). **e** Secondary analysis of microarray profiling of human postmortem substantia nigra samples from patients with PD or healthy controls (GSE26927). *Z*-scores and corrected *p* values were calculated for necroptosis pathway components indicated and displayed via heatmap. ns not significant, *p < 0.05, **p < 0.01, ***p < 0.001. Bars represent group means. *n* = 6 independent replicates in (**b**). *n* = 3 independent replicates in (**c**, **d**).

To confirm that RIPK signaling was also required for functional indications of astrocyte activation, we treated midbrain astrocytes with PFFs or PBS control for 24 h following 30 min pretreatment with RIPK1, RIPK3, or MLKL inhibitors. We then treated differentiated cultures of SH-SY5Y cells with the ACM from these astrocyte cultures at a 1:1 ratio with a normal culture medium (Fig. 7a). As expected, ACM derived from PFF-treated astrocytes greatly reduced the viability of SH-SY5Y cultures. However, this effect could be blocked by inhibition of either RIPK1 or RIPK3, but not MLKL or caspase signaling, in astrocytes (Fig. 7b and Supplementary Fig. 8b, c). We next assessed whether RIPK3 activation downstream of PFFs influenced astrocyte phagocytic activity. To do so, we labeled differentiated SH-SY5Y cultures with CSFE, then used these cultures to generate neuronal debris by subjecting cells to rapid freeze-thaw lysis. We then treated astrocytes with PFFs or PBS control following pretreatment with RIPK3 or NF-κB inhibitors. Following PFF treatment, we exposed astrocytes to CSFE-labeled neuronal debris for 24 h and measured uptake via flow cytometric analysis. PFF treatment significantly reduced uptake of neuronal debris, as measured by geometric mean fluorescence intensity (GMFI) of CSFE within astrocytes (Fig. 7c, d). However, blockade of either RIPK3 or NF-κB completely abolished this effect, confirming that both molecules are required for downregulation of phagocytic activity following exposure to PFFs.

These data suggest that necroptosis-independent RIPK activity engages both transcriptional and functional activation of astrocytes following exposure to fibrillar α-synuclein. We thus returned to our secondary analysis of gene expression in the substantia nigra of Parkinson’s patients in order to see if there was evidence of increased expression of this pathway in human PD. We observed significant upregulation of *RIPK3* in PD patients compared to normal controls, while expression of both *RIPK1* and *MLKL* did not reach statistical significance (Fig. 7e). Together, these data identify a previously unknown function for the RIPKs in the promotion of a neurotoxic activation state and suggest further work is needed to identify roles for necroptosis-independent RIPK signaling in Parkinsonian neurodegeneration and other synucleinopathies.

## Discussion

Abnormal aggregation of α-synuclein is a pathological hallmark of PD [52–54]. However, the effects of pathogenic α-synuclein species on astrocytes have not been thoroughly studied. Previous work has shown that astrocytes can protect dopaminergic neurons from α-synuclein deposition and degeneration [55]. However, α-synuclein aggregates are also frequently observed in PD patients, where they disturb vital homeostatic functions and thereby exacerbate disease pathology in neurons [56]. We show that α-synuclein PFFs induce a potently inflammatory transcriptional program in human midbrain astrocytes that is associated with neurotoxic activity and decreased homeostatic phagocytic function. Notably, this effect requires RIPK signaling, but does not result in necroptosis, the canonical function of this pathway. RIPK3 activation in astrocytes has been observed in mouse models of PD [28], as well as postmortem human patient samples [27]. Moreover, both genetic and pharmacological ablation of RIPK3 signaling have been shown to be protective in mouse models of neurodegeneration [57, 58]. Our findings suggest that therapies targeting RIPK signaling may serve to limit the pathologic consequences of inflammatory astrocyte activation in PD and other neurodegenerative diseases.

Since the initial description of the A1 and A2 astrocyte subtypes, transcriptional profiling of astrocytes as an indication of their A1 or A2 activation state has grown increasingly popular, though whether the respective transcriptomes of these states are stable across disease stimuli or reliably indicate neurotoxic vs. neurotrophic functional activity is controversial [17, 59]. In our study, treatment with α-synuclein PFFs induced neurotoxic activity and diminished phagocytic capacity, in line with previous descriptions of the A1 activation state [18, 60]. However, while α-synuclein PFFs induced many putative A1-associated genes, we also saw marked upregulation of A2-associated genes as well, though each gene set did display somewhat different expression kinetics. Together, our data suggest that expression of both A1 and A2 genes is associated with clear functional changes in neurotoxic and phagocytic activity following exposure to α-synuclein PFFs, supporting other recent work demonstrating that gene expression profiling alone is insufficient to predict the functional outcomes of astrocyte activation [59].

RIPK1 and RIPK3 are activators of necroptotic cell death [61, 62], which has been shown to exert complex functions in diverse disease states, including infection [39, 63, 64], cancer [65], and sterile injury [66]. In the CNS, RIPK signaling has principally been connected to deleterious neuroinflammation and necroptosis in the context of neurodegenerative diseases, including PD [67, 68]. However, we and others recently described a necroptosis-independent function for RIPK signaling in neurons in which a RIPK-dependent transcriptional program induced protective neuroinflammation in response to viral infection [33, 34]. Here, we show that necroptosis-independent RIPK signaling can also coordinate inflammatory transcription in astrocytes, which is associated with the conferral of neurotoxic activity. These findings suggest that cell death-independent functions of this pathway in the CNS may extend beyond neurons, possibly to all cells of neuroectodermal lineage.

While we have identified a previously unknown function for RIPK signaling and NF-κB in promoting a neurotoxic astrocyte activation state, several questions remain. For example, the mechanisms by which aggregated α-synuclein is sensed by astrocytes prior to the induction of inflammatory activation is poorly understood. While some studies have suggested that α-synuclein aggregates engage TLR4-mediated innate sensing pathways [23], whether adult astrocytes express TLR4 in vivo is a matter of debate [18, 69, 70]. Further work, particularly in rodent models, will help further refine our understanding of the upstream signaling events that promote RIPK3 activation in astrocytes in the context of synucleinopathy. Finally, while several groups have now shown that activated astrocytes express some secreted factor that exerts neurotoxic activity, the identity of this factor (or factors) and its mechanism of action have been difficult to discern [16]. Ongoing work characterizing the secretomes of activated astrocytes is needed to answer these important questions.

## Supplementary information

Supplementary Information

## Data Availability

Microarray results are derived from secondary analysis of two previously published datasets, which can be accessed via NCBI’s Gene Expression Omnibus using accession numbers GSE11574 and GSE26927. Raw data from this study are available upon reasonable request to the corresponding author.

## References

[1] Blaszczyk JW (2016). Parkinson’s disease and neurodegeneration: GABA-collapse hypothesis. Front Neurosci..

[2] Scherman D, Desnos C, Darchen F, Pollak P, Javoy-Agid F, Agid Y (1989). Striatal dopamine deficiency in Parkinson’s disease: Role of aging. Ann Neurol..

[3] Braak H, Rub U, Gai WP, Del Tredici K (2003). Idiopathic Parkinson’s disease: possible routes by which vulnerable neuronal types may be subject to neuroinvasion by an unknown pathogen. J Neural Transm..

[4] Wakabayashi K, Tanji K, Mori F, Takahashi H (2007). The Lewy body in Parkinson’s disease: Molecules implicated in the formation and degradation of alpha-synuclein aggregates. Neuropathology.

[5] McCann H, Stevens CH, Cartwright H, Halliday GM (2014). alpha-Synucleinopathy phenotypes. Parkinsonism Relat Disord..

[6] Poewe W, Seppi K, Tanner CM, Halliday GM, Brundin P, Volkmann J (2017). Parkinson disease. Nat Rev Dis Prim..

[7] Sofroniew MV, Vinters HV (2010). Astrocytes: biology and pathology. Acta Neuropathol..

[8] Yun SP, Kam TI, Panicker N, Kim S, Oh Y, Park JS (2018). Block of A1 astrocyte conversion by microglia is neuroprotective in models of Parkinson’s disease. Nat Med.

[9] Kam TI, Hinkle JT, Dawson TM, Dawson VL (2020). Microglia and astrocyte dysfunction in Parkinson’s disease. Neurobiol Dis..

[10] Chun H, Lee CJ (2018). Reactive astrocytes in Alzheimer’s disease: A double-edged sword. Neurosci Res.

[11] Diaz-Castro B, Gangwani MR, Yu X, Coppola G, Khakh BS (2019). Astrocyte molecular signatures in Huntington’s disease. Sci Transl Med.

[12] Tripathi P, Rodriguez-Muela N, Klim JR, de Boer AS, Agrawal S, Sandoe J (2017). Reactive astrocytes promote ALS-like degeneration and intracellular protein aggregation in human motor neurons by disrupting autophagy through TGF-beta1. Stem Cell Rep..

[13] Yamanaka K, Komine O (2018). The multi-dimensional roles of astrocytes in ALS. Neurosci Res.

[14] Pekny M, Wilhelmsson U, Pekna M (2014). The dual role of astrocyte activation and reactive gliosis. Neurosci Lett..

[15] Li K, Li J, Zheng J, Qin S (2019). Reactive astrocytes in neurodegenerative diseases. Aging Dis..

[16] Liddelow SA, Barres BA (2017). Reactive astrocytes: production, function, and therapeutic potential. Immunity.

[17] Escartin C, Guillemaud O, Carrillo-de Sauvage MA (2019). Questions and (some) answers on reactive astrocytes. Glia.

[18] Liddelow SA, Guttenplan KA, Clarke LE, Bennett FC, Bohlen CJ, Schirmer L (2017). Neurotoxic reactive astrocytes are induced by activated microglia. Nature.

[19] Phatnani H, Maniatis T (2015). Astrocytes in neurodegenerative disease. Cold Spring Harb Perspect Biol.

[20] Wang Q, Liu Y, Zhou J (2015). Neuroinflammation in Parkinson’s disease and its potential as therapeutic target. Transl Neurodegener..

[21] Hirsch EC, Hunot S (2009). Neuroinflammation in Parkinson’s disease: a target for neuroprotection?. Lancet Neurol..

[22] Tansey MG, Goldberg MS (2010). Neuroinflammation in Parkinson’s disease: Its role in neuronal death and implications for therapeutic intervention. Neurobiol Dis..

[23] Rannikko EH, Weber SS, Kahle PJ (2015). Exogenous alpha-synuclein induces toll-like receptor 4 dependent inflammatory responses in astrocytes. BMC Neurosci..

[24] Lee HJ, Suk JE, Patrick C, Bae EJ, Cho JH, Rho S (2010). Direct transfer of alpha-synuclein from neuron to astroglia causes inflammatory responses in synucleinopathies. J Biol Chem..

[25] Du RH, Zhou Y, Xia ML, Lu M, Ding JH, Hu G (2018). alpha-Synuclein disrupts the anti-inflammatory role of Drd2 via interfering beta-arrestin2-TAB1 interaction in astrocytes. J Neuroinflammation.

[26] Dionisio PA, Oliveira SR, Gaspar MM, Gama MJ, Castro-Caldas M, Amaral JD (2019). Ablation of RIP3 protects from dopaminergic neurodegeneration in experimental Parkinson’s disease. Cell Death Dis..

[27] Onate M, Catenaccio A, Salvadores N, Saquel C, Martinez A, Moreno-Gonzalez I (2020). The necroptosis machinery mediates axonal degeneration in a model of Parkinson disease. Cell Death Differ..

[28] Iannielli A, Bido S, Folladori L, Segnali A, Cancellieri C, Maresca A (2018). Pharmacological inhibition of necroptosis protects from dopaminergic neuronal cell death in Parkinson’s disease models. Cell Rep..

[29] Tatton WG, Chalmers-Redman R, Brown D, Tatton N (2003). Apoptosis in Parkinson’s disease: Signals for neuronal degradation. Ann Neurol..

[30] Andreone BJ, Larhammar M, Lewcock JW (2020). Cell death and neurodegeneration. Cold Spring Harb Perspect Biol.

[31] Galluzzi L, Kepp O, Chan FK, Kroemer G (2017). Necroptosis: mechanisms and relevance to disease. Annu Rev Pathol..

[32] Shan B, Pan H, Najafov A, Yuan J (2018). Necroptosis in development and diseases. Genes Dev.

[33] Daniels BP, Snyder AG, Olsen TM, Orozco S, Oguin TH, Tait SWG (2017). RIPK3 restricts viral pathogenesis via cell death-independent neuroinflammation. Cell.

[34] Daniels BP, Kofman SB, Smith JR, Norris GT, Snyder AG, Kolb JP (2019). The nucleotide sensor ZBP1 and kinase RIPK3 induce the enzyme IRG1 to promote an antiviral metabolic state in neurons. Immunity.

[35] Najjar M, Saleh D, Zelic M, Nogusa S, Shah S, Tai A (2016). RIPK1 and RIPK3 kinases promote cell-death-independent inflammation by Toll-like receptor 4. Immunity.

[36] Moriwaki K, Balaji S, Bertin J, Gough PJ, Chan FK (2017). Distinct kinase-independent role of RIPK3 in CD11c(+) mononuclear phagocytes in cytokine-induced tissue repair. Cell Rep..

[37] Moriwaki K, Chan FK (2017). The inflammatory signal adaptor RIPK3: functions beyond necroptosis. Int Rev Cell Mol Biol..

[38] Hanggi K, Vasilikos L, Valls AF, Yerbes R, Knop J, Spilgies LM (2017). RIPK1/RIPK3 promotes vascular permeability to allow tumor cell extravasation independent of its necroptotic function. Cell Death Dis..

[39] Nogusa S, Thapa RJ, Dillon CP, Liedmann S, Oguin TH, Ingram JP (2016). RIPK3 activates parallel pathways of MLKL-driven necroptosis and FADD-mediated apoptosis to protect against influenza A virus. Cell Host Microbe.

[40] Daniels BP, Oberst A. Outcomes of RIP kinase signaling during neuroinvasive viral infection. Curr Top Microbiol Immunol. 2020. https://link.springer.com/chapter/10.1007%2F82_2020_204#citeas. 10.1007/82_2020_204 PMC7781604 32253569

[41] Polinski NK, Volpicelli-Daley LA, Sortwell CE, Luk KC, Cremades N, Gottler LM (2018). Best practices for generating and using alpha-synuclein pre-formed fibrils to model Parkinson’s disease in rodents. J Parkinsons Dis..

[42] Wordehoff MM, Hoyer W (2018). Alpha-Synuclein aggregation monitored by thioflavin T fluorescence assay. Bio Protoc.

[43] Mi H, Muruganujan A, Ebert D, Huang X, Thomas PD (2019). PANTHER version 14: more genomes, a new PANTHER GO-slim and improvements in enrichment analysis tools. Nucleic Acids Res.

[44] Wu Q, Takano H, Riddle DM, Trojanowski JQ, Coulter DA, Lee VM (2019). alpha-Synuclein (alphaSyn) preformed fibrils induce endogenous alphaSyn aggregation, compromise synaptic activity and enhance synapse loss in cultured excitatory hippocampal neurons. J Neurosci..

[45] Volpicelli-Daley LA, Luk KC, Lee VM (2014). Addition of exogenous alpha-synuclein preformed fibrils to primary neuronal cultures to seed recruitment of endogenous alpha-synuclein to Lewy body and Lewy neurite-like aggregates. Nat Protoc..

[46] Durrenberger PF, Fernando FS, Kashefi SN, Bonnert TP, Seilhean D, Nait-Oumesmar B (2015). Common mechanisms in neurodegeneration and neuroinflammation: a BrainNet Europe gene expression microarray study. J Neural Transm..

[47] Hu YB, Zhang YF, Wang H, Ren RJ, Cui HL, Huang WY (2019). miR-425 deficiency promotes necroptosis and dopaminergic neurodegeneration in Parkinson’s disease. Cell Death Dis..

[48] Liu J, Zhu Z, Wang L, Du J, Zhang B, Feng X (2020). Functional suppression of Ripk1 blocks the NF-kappaB signaling pathway and induces neuron autophagy after traumatic brain injury. Mol Cell Biochem.

[49] Yatim N, Jusforgues-Saklani H, Orozco S, Schulz O, Barreira da Silva R, Reis e Sousa C (2015). RIPK1 and NF-kappaB signaling in dying cells determines cross-priming of CD8(+) T cells. Science.

[50] Snyder AG, Hubbard NW, Messmer MN, Kofman SB, Hagan CE, Orozco SL (2019). Intratumoral activation of the necroptotic pathway components RIPK1 and RIPK3 potentiates antitumor immunity. Sci Immunol.

[51] Newton K, Sun X, Dixit VM (2004). Kinase RIP3 is dispensable for normal NF-kappa Bs, signaling by the B-cell and T-cell receptors, tumor necrosis factor receptor 1, and Toll-like receptors 2 and 4. Mol Cell Biol..

[52] Gibb WR, Lees AJ (1991). Anatomy, pigmentation, ventral and dorsal subpopulations of the substantia nigra, and differential cell death in Parkinson’s disease. J Neurol Neurosurg Psychiatry.

[53] Spillantini MG, Schmidt ML, Lee VM-Y, Trojanowski JQ, Jakes R, Goedert M (1997). α-Synuclein in Lewy bodies. Nature.

[54] Goedert M, Spillantini MG, Del Tredici K, Braak H (2013). 100 years of Lewy pathology. Nat Rev Neurol..

[55] Tsunemi T, Ishiguro Y, Yoroisaka A, Valdez C, Miyamoto K, Ishikawa K (2020). Astrocytes protect human dopaminergic neurons from α-Synuclein accumulation and propagation. J Neurosci.

[56] Sorrentino ZA, Giasson BI, Chakrabarty P (2019). alpha-Synuclein and astrocytes: tracing the pathways from homeostasis to neurodegeneration in Lewy body disease. Acta Neuropathol..

[57] Ofengeim D, Mazzitelli S, Ito Y, DeWitt JP, Mifflin L, Zou C (2017). RIPK1 mediates a disease-associated microglial response in Alzheimer’s disease. Proc Natl Acad Sci USA.

[58] Ito Y, Ofengeim D, Najafov A, Das S, Saberi S, Li Y (2016). RIPK1 mediates axonal degeneration by promoting inflammation and necroptosis in ALS. Science.

[59] Escartin C, Galea E, Lakatos A, O’Callaghan JP, Petzold GC, Serrano-Pozo A (2021). Reactive astrocyte nomenclature, definitions, and future directions. Nat Neurosci..

[60] Hinkle JT, Dawson VL, Dawson TM (2019). The A1 astrocyte paradigm: new avenues for pharmacological intervention in neurodegeneration. Mov Disord..

[61] Cho Y, Challa S, Moquin D, Genga R, Ray TD, Guildford M (2009). Phosphorylation-driven assembly of the RIP1-RIP3 complex regulates programmed necrosis and virus-induced inflammation. Cell.

[62] Wang H, Sun L, Su L, Rizo J, Liu L, Wang L-F (2014). Mixed lineage kinase domain-like protein MLKL causes necrotic membrane disruption upon phosphorylation by RIP3. Mol Cell.

[63] Upton JW, Kaiser WJ, Mocarski ES (2010). Virus inhibition of RIP3-dependent necrosis. Cell Host Microbe.

[64] Huang Z, Wu S-Q, Liang Y, Zhou X, Chen W, Li L (2015). RIP1/RIP3 binding to HSV-1 ICP6 initiates necroptosis to restrict virus propagation in mice. Cell Host Microbe.

[65] Messmer MN, Snyder AG, Oberst A (2019). Comparing the effects of different cell death programs in tumor progression and immunotherapy. Cell Death Differ..

[66] Zhao H, Jaffer T, Eguchi S, Wang Z, Linkermann A, Ma D (2015). Role of necroptosis in the pathogenesis of solid organ injury. Cell Death Dis..

[67] Yuan J, Amin P, Ofengeim D (2019). Necroptosis and RIPK1-mediated neuroinflammation in CNS diseases. Nat Rev Neurosci..

[68] Oñate M, Catenaccio A, Salvadores N, Saquel C, Martinez A, Moreno-Gonzalez I (2019). The necroptosis machinery mediates axonal degeneration in a model of Parkinson disease. Cell Death Differ.

[69] Zhang Y, Sloan SA, Clarke LE, Caneda C, Plaza CA, Blumenthal PD (2016). Purification and characterization of progenitor and mature human astrocytes reveals transcriptional and functional differences with mouse. Neuron.

[70] Zhang Y, Chen K, Sloan SA, Bennett ML, Scholze AR, O’Keeffe S (2014). An RNA-sequencing transcriptome and splicing database of glia, neurons, and vascular cells of the cerebral cortex. J Neurosci..

