# Supplementary material for: Fibrillar α-synuclein induces neurotoxic astrocyte activation via RIP kinase signaling and NF-κB
Source: Cell Death Dis. 2021 Jul 31;12(8):756. doi: 10.1038/s41419-021-04049-0 (PMC8325686; doi:10.1038/s41419-021-04049-0)
Supplement: Supplementary file 1 — Supplementary Information [file 41419_2021_4049_MOESM1_ESM.pdf]

Supplementary Figures

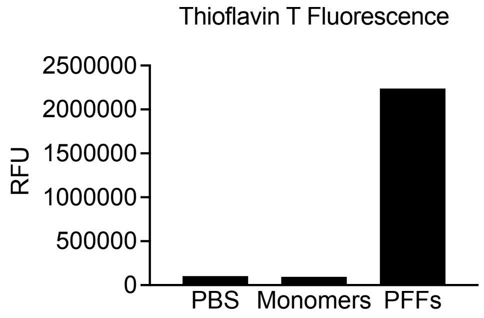

Figure S1. Confirmation of amyloid fibril formation in PFF stocks.
Stocks of α-synuclein monomers and lab-generated PFFs were assessed for fluorescence in the presence of thioflavin T. Increased fluorescence of PFF vs. monomeric stocks (~24 fold) is indicative of successful amyloid fibril formation. See materials and methods for additional details.

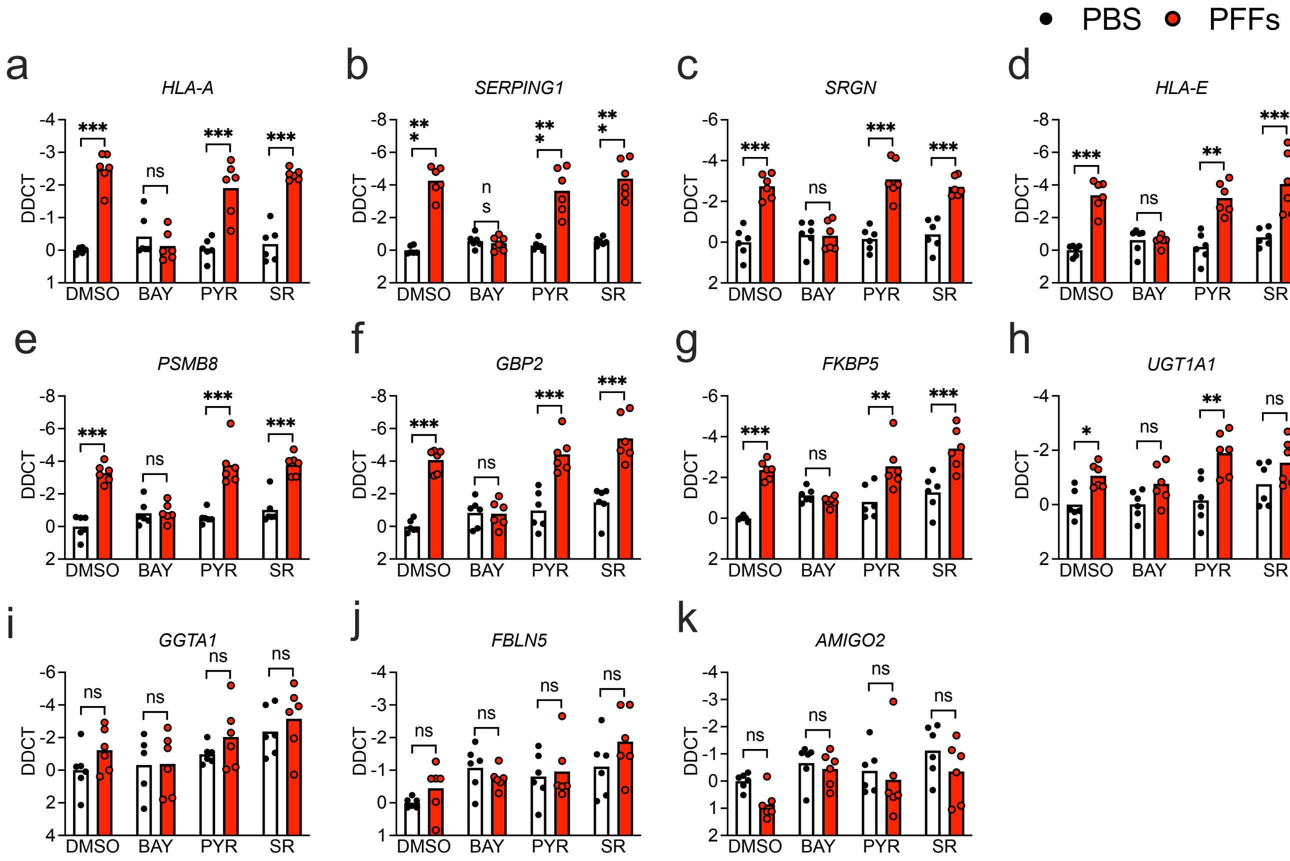

Figure S2. α-synuclein PFFs induce NF-κB-dependent transcriptional activation of A1-associated genes in human midbrain astrocytes.

a-k) Primary human midbrain astrocyte cultures were treated for 24h with PFFs or PBS control solution. Cultures were pretreated (30min) with inhibitors of NF-κB (BAY), JAK/STAT (PYR), or AP1 (SR) signaling prior to addition of PFFs. Levels of indicated A1-associated transcripts were measured using qRT-PCR. ns: not significant, *$p < 0.05$, **$p < 0.01$, ***$p < 0.001$. Bars represent group means. n=6 independent replicates for all experiments.

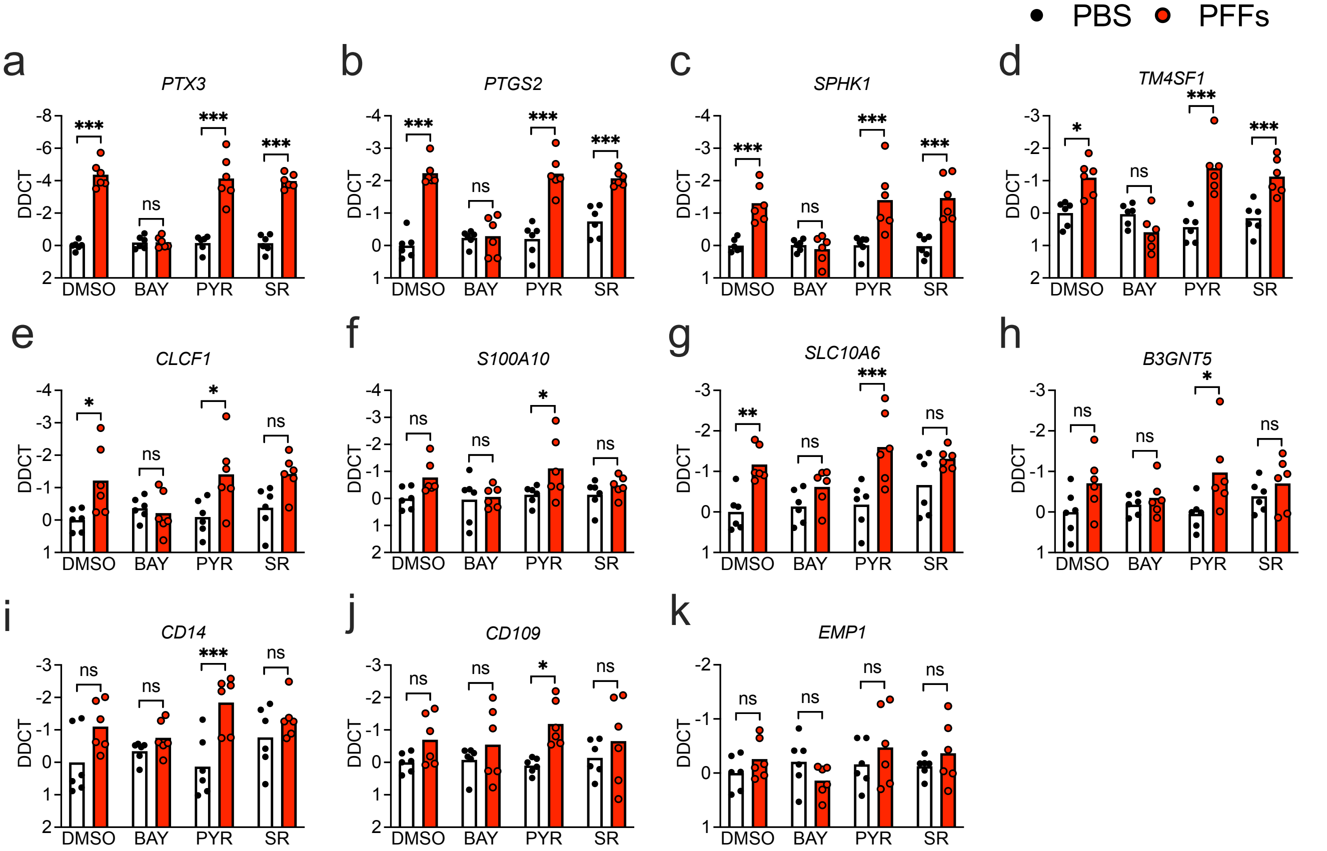

Figure S3. α-synuclein PFFs induce NF-κB-dependent transcriptional activation of A2-associated genes in human midbrain astrocytes.

a-k) Primary human midbrain astrocyte cultures were treated for 24h with PFFs or PBS control solution. Cultures were pretreated (30min) with inhibitors of NF-κB (BAY), JAK/STAT (PYR), or AP1 (SR) signaling prior to addition of PFFs. Levels of indicated A2-associated transcripts were measured using qRT-PCR. ns: not significant, *$p < 0.05$, **$p < 0.01$, ***$p < 0.001$. Bars represent group means. n=6 independent replicates for all experiments.

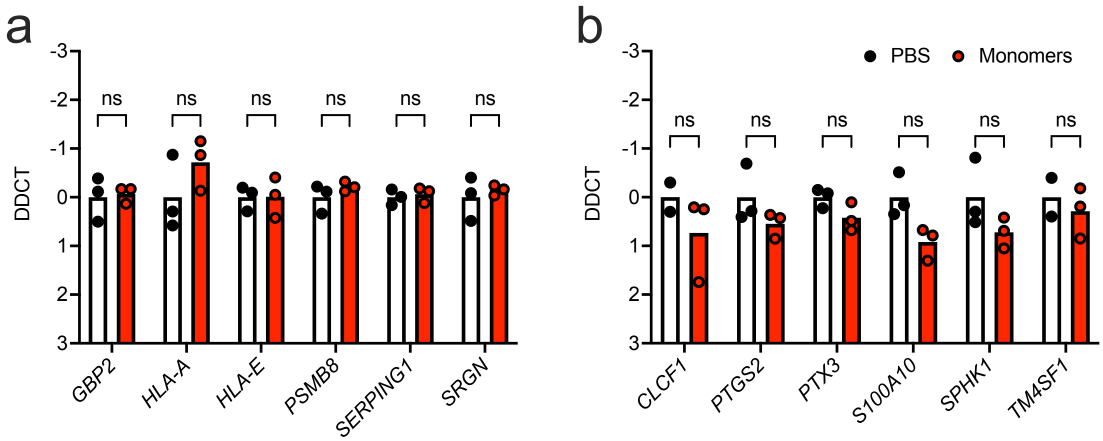

Figure S4. α-synuclein monomers do not induce gene expression associated with astrocyte activation. **a-b)** Primary human midbrain astrocyte cultures were treated for 24h with α-synuclein monomers or PBS control solution. Levels of indicated A1- (a) or A2- (b) associated transcripts were measured using qRT-PCR. ns: not significant. Bars represent group means. n=3 independent replicates for all experiments.

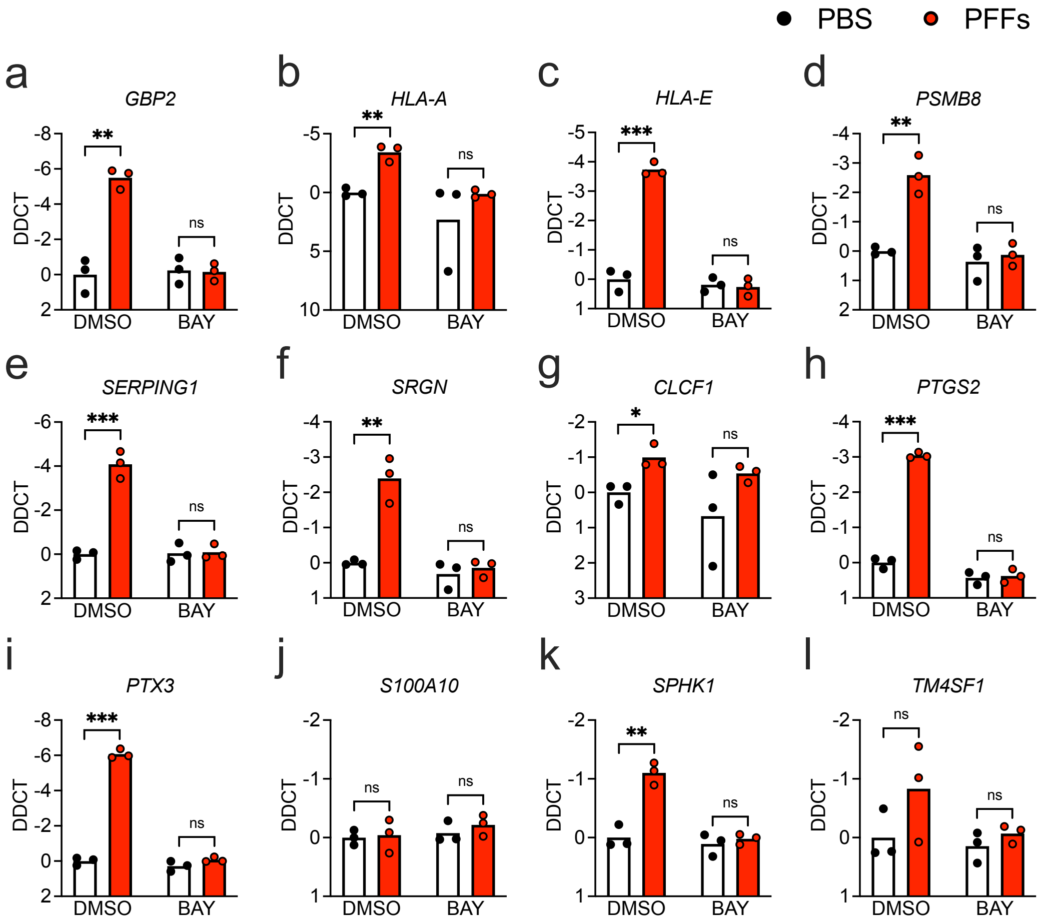

Figure S5. PFFs induce NF-κB-dependent transcriptional activation in human midbrain astrocytes maintained in serum-free medium.

a-l) Primary human midbrain astrocyte cultures maintained in serum-free medium were treated for 24h with PFFs or PBS control solution. Cultures were pretreated (30min) with an inhibitor of NF-κB (BAY) prior to addition of PFFs**.** Levels of indicated A1- (a-f) and A2- (g-l) associated transcripts were measured using qRT-PCR. ns: not significant, *$p < 0.05$, **$p < 0.01$, ***$p < 0.001$. Bars represent group means. n=3 independent replicates for all experiments.

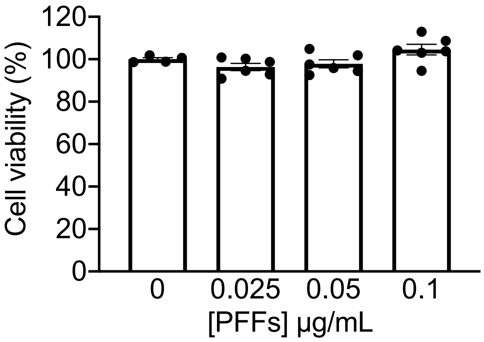

Figure S6. PFFs are not toxic to differentiated SH-SY5Y cultures.
Differentiated cultures of SH-SY5Y cells were treated with the indicated concentrations of PFFs for 24h. Cell viability was then assessed using an ATP-luciferase assay (CellTiter Glo). n= 4-6 independent replicates.

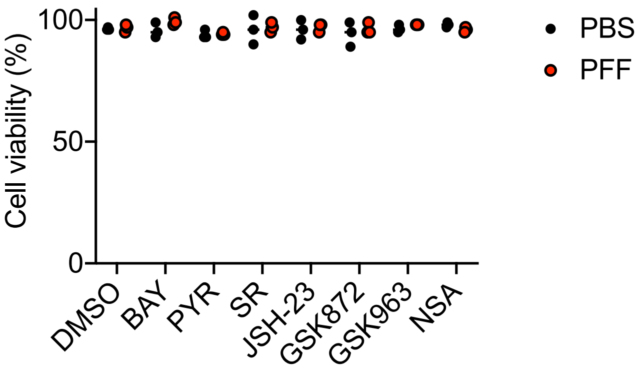

Figure S7. Neither PFFs nor transcription factor/necroptosis pathway inhibitors induce cell death in human midbrain astrocyte cultures.
Primary human midbrain astrocyte cultures were treated with PFFs or PBS along with indicated inhibitors for 24h. Cell viability was then assessed using an ATP-luciferase assay (CellTiter Glo). n= 3 independent replicates.

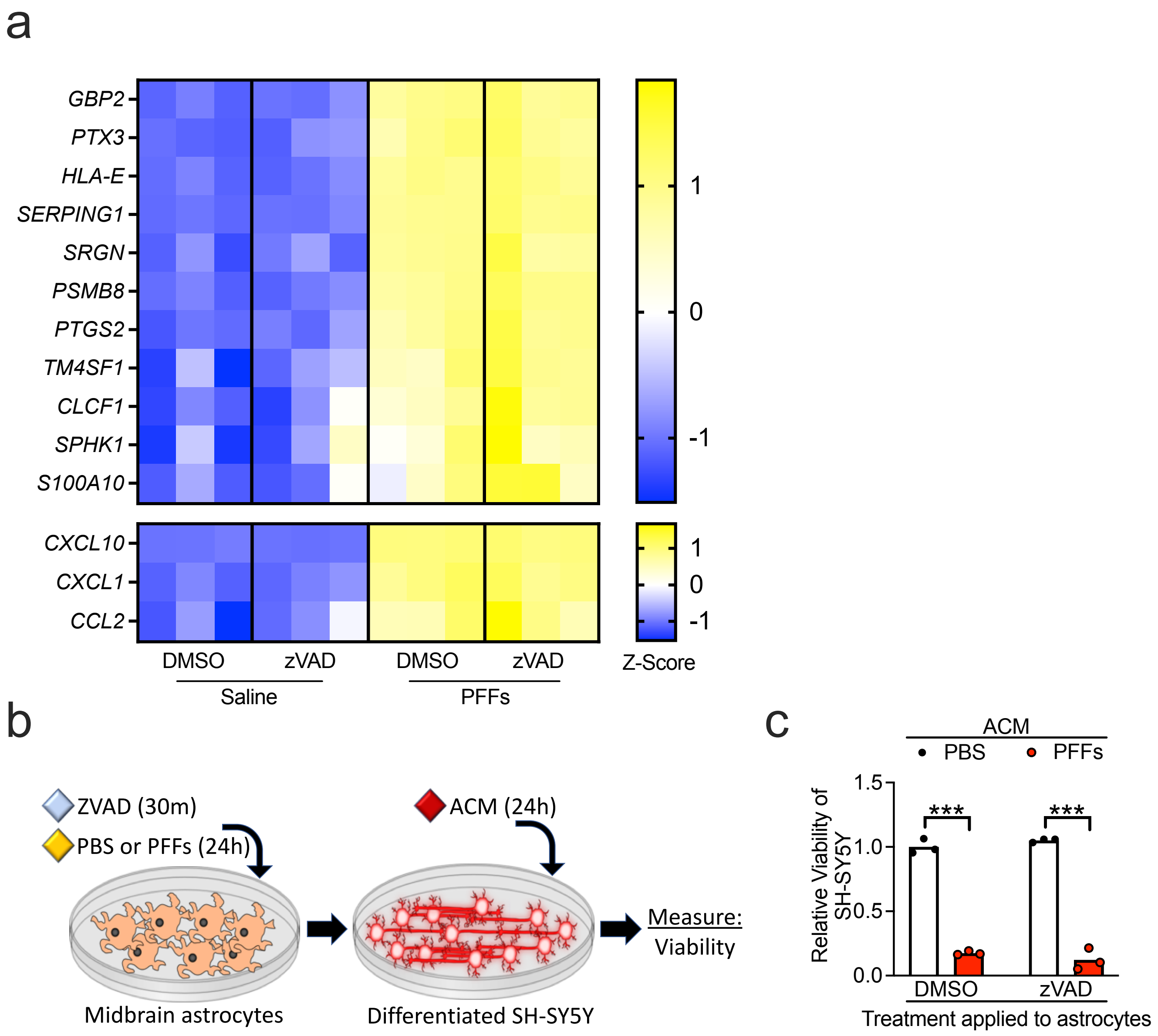

Figure S8. PFF-mediated astrocyte activation does not require caspase signaling.
a) Primary human midbrain astrocyte cultures were treated for 24h with PFFs or PBS control solution. Cultures were pretreated (30min) with an inhibitor of caspase signaling (zVAD) prior to addition of PFFs**.** Levels of indicated transcripts were measured using qRT-PCR. **b-c)** Primary human midbrain astrocytes were treated with zVAD and/or PFFs, as indicated. After 24h, astrocyte conditioned medium (ACM) was applied (1:1) to differentiated SH-SY5Y cultures for 24h followed by endpoint analyses. **c)** Viability of SH-SY5Y cells following treatment with ACM derived from astrocyte cultures treated with the indicated inhibitors was measured via ATP-luciferase assay (Cell Titer Glo). ns: not significant, ***p < 0.001. Bars represent group means. n=3 independent replicates for all experiments.

Gene	Forward	Reverse
18S	AGAAACGGCTACCACATCCA	CCCTCCAATGGATCCTCGTT
AMIGO2	CTTCAGCGTTTGGAGGGCT	CAGGGAACAGTCACAGACAAAT
AXL	CCAGGACACCCCAGAGGTGCTAAT	TGGTGGACTGGCTGTGCTTGC
B3GNT5	ACTCCTCCCCAACAAGGTCT	TTTAACCCCAAACTGGCAAC
CCL2	GCAGCAAGTGTCCCAAAGAA	CTGGGGAAAGCTAGGGGAAA
CD109	CAGGAATGTGGACTCTGGGT	CTTTCGGACATGTGGACTGC
CD14	CCGCTGTGTAGGAAAGAAGC	GCAGCGGAAATCTTCATCGT
CLCF1	GCACAGAGTGGCAAACAAAA	ACACCCCAAAATGCTACTGC
CXCL1	ACTCTACCTGCACACTGTCC	TCCCCTGCCTTCACAATGAT
CXCL10	GTGGCATTCAAGGAGTACCTC	TGATGGCCTTCGATTCTGGATT
EMP1	CCAGTACACCAGCAGAGGAA	AACAGTAGCGATGTGGACCA
FBLN5	TCGCCAGTCAGGACAGTGT	AGTAGGGGTTCGAGTAGGGC
FKBP5	CTCCCTAAAATTCCCTCGAATGC	CCCTCTCCTTTCCGTTTGGTT
GAS6	ATCAAGGTCAACAGGGATGC	CTTCTCCGTTCAGCCAGTTC
GBP2	CTATCTGCAATTACGCAGCCT	TGTTCTGGCTTCTTGGGATGA
GGTA1	ATGACAGCAGTGCTCAGAAGG	AGCCGAAGCTCTGTTGTGT
HLA-A	GACCAGGAGACACGGAATGTG	CCTCGTTCAAGGCGATGTAATC
HLA-E	TTCCGAGTGAATCTGCGGAC	GTCGTAGGCGAACTGTTCATAC
IKBKE	TGCGTGCAGAAGTATCAAGC	TACAGGCAGCCACAGAACAG
LCN2	GAAGTGTGACTACTGGATCAGGA	ACCACTCGGACGAGGTAACT
MEGF10	TGACTGCTTGCCTGGCTTCACA	GTTACAGGTTCCGTTGTTGGTGC
MERTK	CAGGAAGATGGGACCTCTCTGA	GGCTGAAGTCTTTCATGCACGC
NFKB1	GCAGCACTACTTCTTGACCACC	TCTGCTCCTGAGCATTGACGTC
NFKB2	GGCAGACCAGTGTCATTGAGCA	CAGCAGAAAGCTCACCACACTC
PSMB8	GGTCCTACATTAGTGCCTTACGG	CGCAGATAGTACAGCCTGCATT
PTGS2	TGAGCATCTACGGTTTGCTG	TGCTTGTCTGGAACAACTGC
PTX3	GTGGGTGGAGAGGAGAACAA	TTCCTCCCTCAGGAACAATG
S100A10	ATGAAGGACCTGGACCAGTG	GCAGATTCCTTAAGCGACCC
SERPING1	GGGATGCTTTGGTAGATTTCTCC	GAGGATGCTCTCCAGGTTTGT
SLC10A6	TGTTGCCGATGTTCATTTGT	CACTGTGAAAACGAGCTCCA
SPHK1	ACCCATGAACCTGCTGTCTC	CAGGTGTCTTGGAACCCACT
SRGN	GGACTACTCTGGATCAGGCTT	CAAGAGACCTAAGGTTGTCATGG
TM4SF1	AAGGGGGAGAAAACCTAGCA	CCAGCCCAATGAAGACAAAT
TRAF2	CACCGGTACTGCTCCTTCTG	TGAACACAGGCAGCACAGTT
UGT1A1	TGTCCCATGCTGGGAAGATAC	GAATGGCACAGGGTACGTCT

Table S1: Primer sequences for qRT-PCR
